# Impact of changes in gestational diabetes mellitus diagnostic criteria during the COVID-19 pandemic

**DOI:** 10.1007/s11845-025-03926-3

**Published:** 2025-03-12

**Authors:** Jessica Neville, Kelly Foley, Seán Lacey, Antoinette Tuthill, Oratile Kgosidialwa, Mairead O’Riordan, Fiona O’Halloran, Seán J. Costelloe

**Affiliations:** 1 Department of Clinical Biochemistry, Cork University Hospital (CUH), T12 P928 Wilton, Cork Ireland; 2 Department of Biological Science, Munster Technological University, Bishopstown Campus, T12 P928 Cork, Ireland; 3 Research Integrity & Compliance Officer, Munster Technological University, Rossa Ave, Bishopstown, T12 P928 Cork, Ireland; 4 Department of Diabetes and Endocrinology, CUH, T12 P928 Cork, Ireland; 5 Department of Obstetrics and Gynaecology, CUMH, T12 P928 Cork, Ireland

**Keywords:** COVID-19, Diagnostic criteria, Gestational diabetes mellitus, Oral glucose tolerance test

## Abstract

**Background/Aims:**

During the COVID-19 pandemic, the Health Service Executive (HSE) and Royal College of Obstetricians and Gynaecologists (RCOG) recommended fasting and random plasma glucose (FPG/RPG) alongside glycated haemoglobin (HbA_1c_) to replace the oral glucose tolerance test (OGTT) for diagnosing Gestational Diabetes Mellitus (GDM).

**Methods:**

The study compared testing patterns and diagnostic rates for GDM before and after implementing the RCOG guidelines (01/05/2020) in pregnancies beginning 01/11/2018 to 31/03/2021. Trends were inspected using Cochrane-Armitage tests. Differences between General Practice (GP) and Secondary Care (SCare) were assessed by chi-square analysis. A significance level of *p* < 0.05 was used for all analyses. Information on maternal and pregnancy characteristics was accessed where available.

**Results:**

Data indicated a significant reduction in OGTTs requested by GPs and SCare. Conversely, HbA_1c_, FPG and RPG test requests increased significantly in both locations. The overall GDM positivity rate increased significantly from 7.4% to 22.0% in GP and 16.9% to 39.0% in SCare following RCOG guideline implementation.

**Conclusions:**

The RCOG guidelines appear to have been well adopted by GPs and SCare, with greater adherence in SCare. Using FPG, RPG and HbA_1c_ to a greater extent than the OGTT corresponded with increased GDM diagnostic rates. Given the difficulties with interpreting HbA_1c_ in pregnancy, its routine use in diagnosing GDM requires further careful consideration. Relaying changes in diagnostic protocol during pandemics requires strong communication with all requesting clinicians, including GPs. Comparisons between GP and SCare indicated significant differences in test-requesting practices and GDM positivity rates.

## Introduction

Gestational Diabetes Mellitus (GDM) may be defined as carbohydrate intolerance resulting in hyperglycaemia of variable severity with first onset or recognition during pregnancy [1]. The majority of GDM cases are due to impaired glucose tolerance caused by the dysfunction of pancreatic β-cells [2]. GDM is one of the most common complications of pregnancy and can affect one in six pregnancies. Globally, the prevalence of GDM is 14%, while in Europe, GDM is estimated to be slightly lower at approximately 11% [3]. GDM risk factors include high body mass index (BMI), advanced maternal age, and a family history of diabetes [4] [5]. GDM is associated with pre-eclampsia, fetal macrosomia, birth injury, neonatal shoulder dystocia and neonatal hypoglycemia in the short term [6] and Type 2 Diabetes Mellitus (T2DM) in the long term, which can require lifelong clinical management [7].

The SARS-CoV-2 (COVID-19) viral pandemic was officially declared by the World Health Organization (WHO) on 11/03/2020 and had a profound impact on health services globally [8]. The guidance regarding social distancing, hand hygiene, self-isolation and other measures to limit the spread of the COVID-19 virus impacted everyone in society, particularly people who were reliant on specific health-related support systems, such as pregnant women and those with non-communicable diseases, including diabetes [9]. The pandemic caused the curtailment of outpatient appointments, postponement of routine medical visits and redeployment of healthcare staff to the pandemic response. All these measures directly impacted patient care, including antenatal care and diabetes services. Pregnancy induces natural physiological changes that can lead to adaptations of the immune and cardiopulmonary systems, which can make pregnant women more prone to developing severe illness after infection with respiratory viruses, particularly in the third trimester of pregnancy [10]. Thus, to reduce the risk of pregnant women contracting COVID-19, all aspects of patient care were reconsidered, including GDM screening, diagnosis, and management.

In Ireland, GDM is screened for with an oral glucose tolerance test (OGTT), usually in the second trimester of pregnancy (24–28 weeks’ gestation) [11]. It is estimated that Cork University Maternity Hospital (CUMH) performs approximately 1200 OGTTs, with an estimated 400 additional OGTTs performed by general practitioners (GPs) in primary care in the region [12]. CUMH adopts a risk factor based screening strategy for GDM. Risk factors include family history of diabetes, body mass index (BMI) greater than 30, woman aged greater than 40, had a previous unexplained stillbirth, glycosuria, previously given birth to a baby weighing greater than 4.5 kg, have polycystic ovary syndrome (PCOS) and have polyhydramnios The OGTT test assesses the ability of a patient to respond to a large volume of glucose. After an overnight fast, blood is initially taken to determine fasting blood glucose levels. The patient is then given a concentrated glucose (75 g) solution to drink; blood is drawn at 1 and 2 h post-consumption, and plasma glucose concentrations are measured [13]. This test requires a patient to spend at least two hours in the hospital, per WHO 2013 criteria [14]. During the COVID-19 pandemic, the WHO identified pregnant women as being at high risk of severe illness if infected with COVID-19, and thus, it was advised that pregnant women should limit time spent in hospital [15].

A diabetes diagnosis during the pandemic was an anxious time for any patient as diabetes, obesity and hypertension were identified as co-morbidities associated with worse outcomes from COVID-19 infection [16]. In response, the Irish Health Service Executive (HSE) adopted procedures recommended by the Royal College of Obstetrics and Gynecologists (RCOG) in the UK to help limit non-urgent hospital visits and reduce hospital footfall during the COVID-19 pandemic [17]. RCOG recommendations included an alternative testing strategy for screening pregnant women for GDM that focused on replacing the 2-h OGTT with other tests of shorter duration. A comparison of the changes made to testing patterns and diagnostic cut-off values for tests following RCOG recommendations is given in Table 1. Recommendations included using HbA_1c_, fasting plasma glucose (FPG) and random plasma glucose (RPG) tests for GDM screening. At the antenatal booking visit (trimester 1 where pregnancy is between 8 and 12 weeks gestation), HbA_1c_ and RPG were used to screen all women at risk of glucose intolerance. A diagnosis of T2DM was made when at least one of the following criteria was met: RPG ≥ 11.1 mmol/L; HbA_1c_ ≥ 48 mmol/mol. GDM was diagnosed at the antenatal booking visit if HbA_1c_ was 41–47 mmol/L. A diagnosis of GDM was made if a pregnant woman had any one of FPG ≥ 5.1 mmol/L, RPG ≥ 9.0 mmol/L, or HbA_1c_ 41–47 mmol/mol at the 28-week screening visit [18].

**Table 1:** Comparison of the testing patterns and diagnostic cut-off values for GDM before and during the COVID-19 pandemic based on recommendations from the RCOG

***Test***	***Pre COVID-19 pandemic*** ***Booking Visit CUMH Criteria*** ***(8–12-week gestation)***	***The COVID-19 Pandemic*** ***Booking Visit CUMH Criteria*** ***(8–12-week gestation)***	***RCOG 2020 Guidelines*** ***Booking Visit*** ***(8–12-week gestation)***	***The COVID-19 Pandemic*** ***24–28-week screening CUMH criteria***	***RCOG 2020 Guidelines*** ***24–28-week screening criteria***
75 g OGTT	Time 0 h: ≥ 5.1 mmol/L Time 1 h: 10.0 mmol/L Time 2 h: ≥ 8.5 mmol/L	N/A	N/A	N/A	N/A
FPG	N/A	≥ 5.1 mmol/L	N/A	≥ 5.1 mmol/L	≥ 5.6 mmol/L or Based on resources, clinical capacity and population characteristics ≥ 5.3 mmol/L
RPG	N/A	≥ 11.1 mmol/L should be managed as T2DM	≥ 11.1 mmol/L managed as T2DM 9–11 mmol/L managed as GDM	≥ 9 mmol/L	≥ 9 mmol/L
HbA_1c_	N/A	≥ 48 mmol/mol managed as T2DM 41–47 mmol/mol at booking or with a history of previous GDM should be managed as having GDM	≥ 48 mmol/mol managed as T2DM 41–47 mmol/mol at booking or with a history of previous GDM should be managed as having GDM	≥ 39 mmol/mol	≥ 39 mmol/mol

Abbreviations: *OGTT*, Oral glucose tolerance test; *FPG*, Fasting plasma glucose; *RPG*, Random plasma glucose; *HbA1c*, Haemoglobin A1c; *CUMH, *Cork University Matermity Hospital; *RCOG*, Royal College of Obstetricians and Gynaecologists

The implication of a missed GDM diagnosis has increased risks for both women and their newborns. CUMH is a large maternity hospital in the South of Ireland that delivers approximately 7000 babies annually [19]. It is part of the clinically led network of maternity hospitals/units in Ireland, called the Ireland South Women & Infants Directorate. Recommendations from the RCOG and HSE were implemented in CUMH on 01/05/2020. The current retrospective study investigated the impact of changes made to testing strategies in CUMH during the COVID-19 pandemic on the diagnosis of GDM. This study aimed to determine the extent to which guidelines were implemented and assess the impact of these changes on GDM diagnostic rates and maternal characteristics for GDM in pregnancies.

## Materials and methods

### Research ethics

The study was approved by the Cork Teaching Hospitals Clinical Research Ethics Committee (approval reference ECM 4), and permission was also obtained from the local information governance group (LIGG) in CUMH.

### Setting

A retrospective single-centre study was performed in CUMH, examining data from pregnant women who were investigated for GDM. The study was divided into two parts.

Part one compared the testing patterns used to diagnose GDM in pregnant women before and after the RCOG guidelines were implemented (01/05/2020). For this, 15,758 pregnancies were examined for time period 01/11/2018 to 31/03/2021. Women were excluded if they were previously known to have diabetes mellitus or had tests diagnostic of diabetes mellitus during their pregnancy.

Part two of this study examined if the COVID-19 pandemic influenced the rate of GDM detection. GDM positivity rates were also compared for the same period in General Practice (GP) and secondary care (SCare). To assess if significant differences between the demographics or relevant clinical history and outcomes might affect these rates, a subset analysis was performed comparing pregnancies with GDM and complete gestation before implementation of the RCOG guidelines, with pregnancies positive for GDM beginning after the guidelines were introduced. For this, information relating to maternal, obstetric and pregnancy characteristics was accessed, including age, BMI, race, family history of diabetes and medical history for women diagnosed with GDM. All laboratory data retrieved from the Department of Clinical Biochemistry, Cork University Hospital (CUH), and all electronic medical records (MN-CMS) were pseudonymised and a retrospective analysis was performed.

### Statistical analysis

Data were extracted and evaluated from the relevant hospital databases using IBM® COGNOS software and analysed using R 4.2.2 and RStudio 6.1.524 for Windows (R Core Team, 2023). Additional packages used included *ggplot2 (3.5.1), car (3.1–1), psych (2.3.3)* and *summarytools (1.0.1)* [20–23]. Descriptive statistics were used to assess the demographic characteristics of GDM patients. Continuous data were presented numerically using the mean and standard deviation for normally distributed data and the median and range for skewed data. Categorical variables were presented using frequencies and percentages. Trends in test request frequency and GDM positivity were assessed using the Cochrane-Armitage test. Statistical differences between groups were analysed using Fisher’s Exact or Chi-square tests for categorical data and an independent *t*-test for means of groups of parametric data. All formal statistical test results were interpreted using a 5% significance level.

## Results

**Fig. 1:**
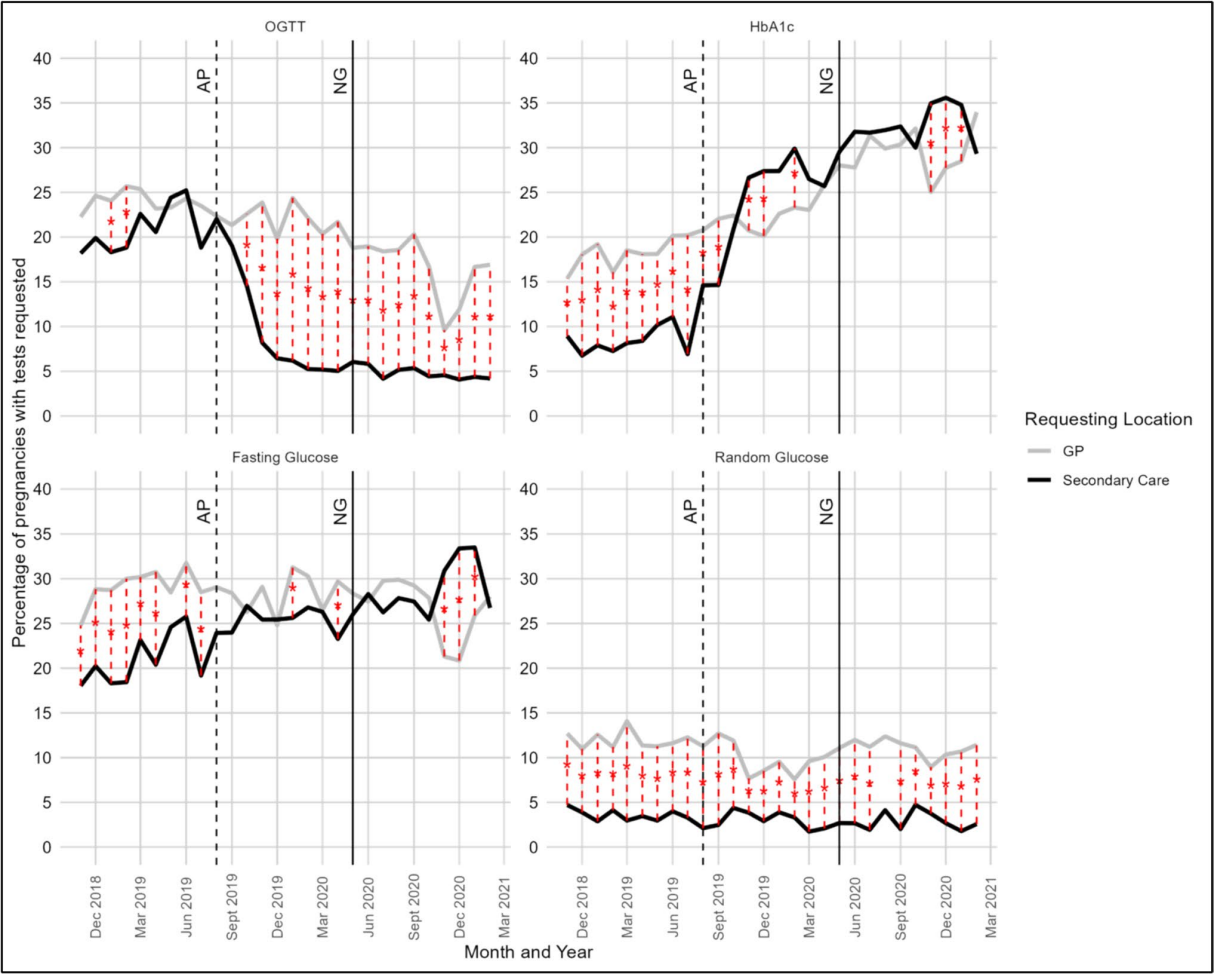
A summary of the percentage of pregnancies that had a Fasting Glucose, Oral Glucose Tolerance Test (OGTT), haemoglobin A1c (HbA_1c_) or Random Glucose test either in General Practice (GP) or from a requesting hospital consultant in secondary care during the time period 01/11/2018 to 31/03/2021.*Abbreviations*: *AP*-Affected pregnancies (All pregnancies from that point forward that either overlap with the new guideline introduction from the Royal college of Obstetricians and Gynaecologists (RCOG) or occur entirely after its introduction) *NG*-New guideline (RCOG new guideline introduction on the 01/05/2020)

Figure 1 compares the tests requesting frequency for GDM screening in GP or SCare test-requesting locations before and after implementing RCOG guidelines in CUMH. A significant reduction in the number of OGTTs requested in both GP (*p* < 0.001) and SCare (*p* < 0.001) settings was observed during this period. Conversely, on average, there was a significant increase in HbA_1c_ test requests in both GP (*p* < 0.001) and SCare settings (*p* < 0.001). However, over the 29 months examined in GP and SCare requesting locations, there was a significant difference in test-requesting patterns for FPG (GP *p* = 0.003, SCare *p* < 0.001) and RPG (GP *p* = 0.036, SCare *p* = 0.015).

**Fig. 2:**
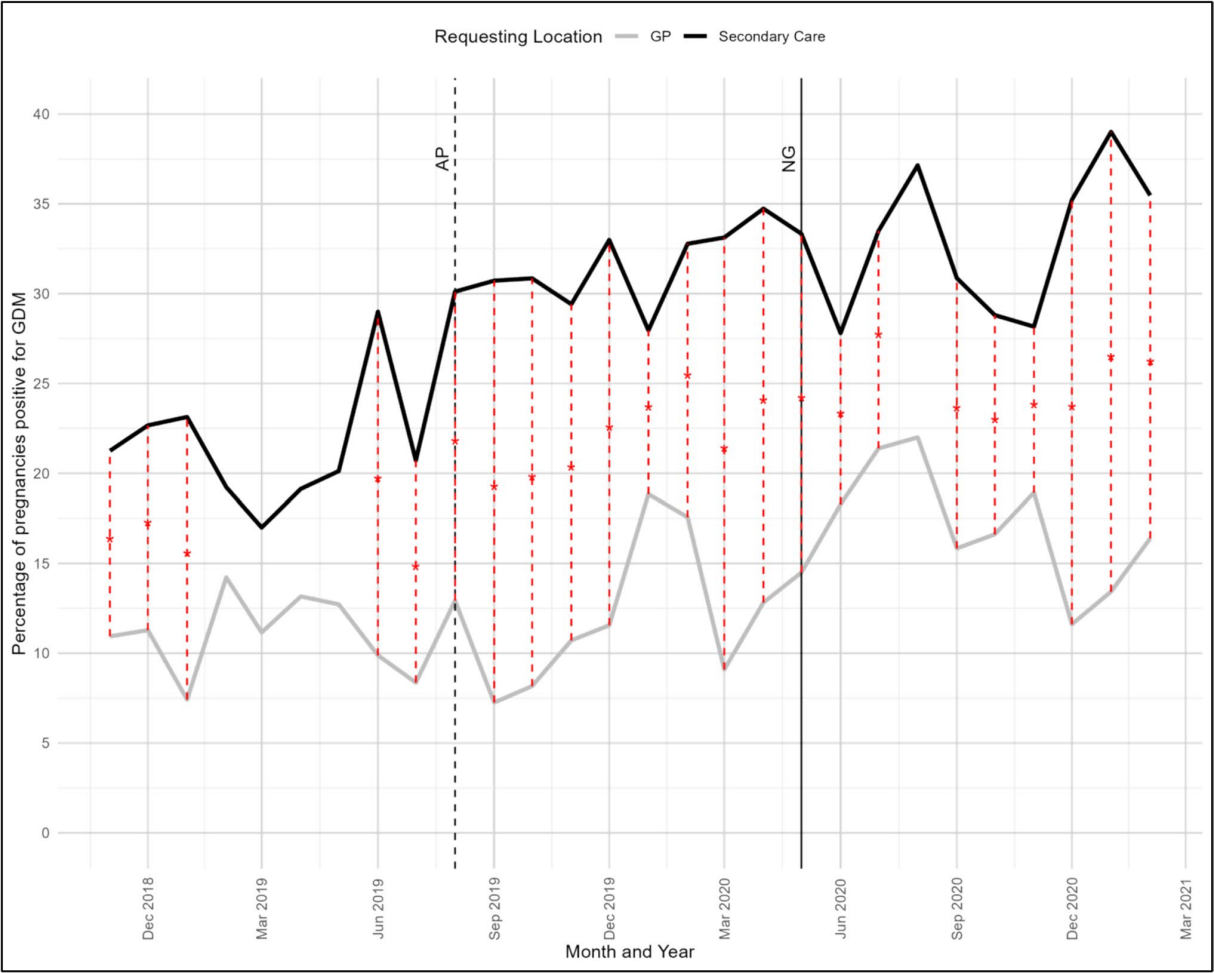
A summary of the percentage of pregnancies that had a positive test for gestational diabetes mellitus (GDM) in General Practice (GP) or Secondary care requesting locations. Abbreviations: AP-Affected pregnancies (All pregnancies from that point forward that either overlap with the new guideline introduction from the Royal college of Obstetricians and Gynaecologists (RCOG) or occur entirely after its introduction) *NG*-New guideline (RCOG new guideline introduction on the 01/05/2020)

The number of pregnancies that had a test positive for GDM in GP or SCare locations was examined over the 29-month time period (Fig. 2). In GP and SCare requesting locations there was a significant difference in the number of pregnancies positive for GDM following the change in guidelines in CUMH recommended by the RCOG (*p* < 0.001). The maximum number of GDM positive pregnancies recorded in GP was 22.0% in August 2020 and in SCare, 39.0% in January 2021. The overall GDM positivity rate increased significantly from 7.4% to 22.0% in GP and 16.9% to 39.0% in SCare following RCOG guideline implementation.

Part two of this study involved investigating the impact of diagnostic changes on the prevalence of GDM and pregnancy outcomes over the study period. There was a significant difference (*p* < 0.001) in the number of women diagnosed with GDM from May to December 2019 (7.5%) compared to the same period in 2020 (12.6%). Of interest, there was no significant difference in live birth rates in CUMH during the study periods, with 4572 live births recorded in 2019 (May to December) and 4320 live births recorded in 2020 (May to December). With regards to pregnancy and maternal characteristics, there were no significant differences in pregnancy characteristics in women diagnosed with GDM (Table 2). However, in GDM positive women with a self-reported history of Polycystic Ovary Syndrome a significant decrease (*p* = 0.002) was observed in Group 2 compared to Group 1.

**Table 2:** Comparison of maternal and pregnancy characteristics in women diagnosed with GDM over two study periods (2019, Group 1 and 2020, Group 2)

Maternal characteristics	Group 1 May–Dec 2019 (***n*** = 343)	Group 2 May–Dec 2020 (***n*** = 548)	***p***-value
Maternal Age (mean ± SD)	34.1 ± 4.92	33.7 ± 4.96	0.207*
BMI kg/m^2^ (mean ± SD) Obese (BMI of > 30 kg/m^2^)	30.8 ± 6.8 52.1%	31.1 ± 6.9 48.5%	0.730*
Race/Ethnicity (%): Irish Irish Traveller Other White background African Chinese Other ethnicity	69.1% 1.4% 13.7% 4.1% 0.4% 11.3%	70.8% 0.9% 13.3% 2.4% 0.4% 12.2%	0.466^
FHx T1DM (%)	3.5%	2.4%	0.306^
FHx T2DM (%)	34.5%	30.8%	0.268^
Hx PCOS (%)	12.6%	6.2%	0.002^
Hypertension (Pre-pregnancy or pregnancy induced) (%)	3.5%	2.4%	0.136^
Macrosomia (> 4.5 kg) (%)	3.2%	3.1%	1.000^

*Abbreviations: FHx T1DM*, Family history of Type 1 Diabetes Mellitus;* FHx T2DM*, Family history Type 2 Diabetes Mellitus;* PCOS*, Polycystic Ovary Syndrome

*^: *Maternal age and BMI p-values were calculated using the t-test

*^Race/Ethnicity, FHx T1DM, FHx T2DM, Hx PCOS, Hypertension and Macrosomia p-values were calculated using the Fishers exact test. *

## Discussion

The COVID-19 pandemic caused major disruptions to global healthcare services and data quantifying this impact is now starting to emerge. The current study assessed the impact of changes in GDM test requesting patterns and diagnosis in an Irish tier-one maternity hospital. A notable shift in the utilisation of screening tests for GDM was clear following the implementation of RCOG guidelines, particularly in terms of OGTTs and HbA_1c_ test requests. Significant changes in test requesting frequency and the percentage of positive GDM cases over time was observed in GP and SCare with no influence on maternal and pregnancy characteristics in GDM pregnancies. Data from the current study indicated that the GDM testing strategy that was applied in CUMH during the COVID-19 pandemic was effective as it identified women most at risk of GDM. Indeed, the increased rate of GDM diagnosis suggests that the change in testing patterns had a positive impact on patient care.

During the COVID-19 pandemic, GDM testing was altered to adhere to social distancing requirements and to reduce the risk of pregnant women being exposed to COVID-19. Pregnant women were identified as being more likely to develop severe illness following infection with respiratory viruses [24] [15]. Diagnosing GDM during the pandemic was a challenge globally and different strategies were taken in different geographical locations. Healthcare services in Ireland and the UK adopted recommendations from the RCOG. Of interest, a report from a tertiary London hospital indicated a decrease in GDM incidence from 7.7% to 4.2% [25], which contrasts with the increased incidence observed in the current study. The authors reported that women who tested negative for GDM using RCOG guidelines were then reassessed with an OGTT at a later stage, and 20% of these were then confirmed as GDM positive [25]. Another Irish study based in the National Maternity Hospital, Dublin, reported a decrease in the rate of positive GDM tests (42.2% in 2019 versus 20% in 2020) [9]. It is worth noting that the study period was shorter and based on data collected over a three-month period in 2019 that was compared to data from the same three months in 2020, following adoption of the RCOG recommendations. The authors concluded that while there was a likely underdiagnosis of GDM, women with greater risk of hyperglycaemia were still correctly identified [9].

A Canadian study reported an underdiagnosis of GDM following the implementation of a strategy that did not use the OGTT as the initial GDM test during the COVID-19 pandemic [24]. Likewise, in Australia, GDM incidence reduced from 25% to 12.7% following a change in GDM screening during the COVID-19 pandemic. In contrast to our study results where an increase in GDM incidence was identified, diagnostic criteria in these studies resulted in a reduction in the frequency of a GDM diagnosis without increased risk in obstetric or neonatal complications of GDM [26]. Another retrospective single-centre study performed in Naples, Italy reported similar results to the current La Verde et al. (2020), a significantly higher incidence of GDM was reported during the lockdown period (9.3%) compared to before the pandemic (3.4%) [27]. In this Italian study, the FPG and HbA_1c_ tests were used for GDM screening instead of the OGTT, with an FPG result of ≥ 5.1 mmol/L deemed acceptable to diagnose GDM, similar to the guidelines adopted in Ireland. This study incorporated a larger study population than those described previously as two cohorts of patients were analysed (ten months prior to lockdown and ten months during or after the lockdown period) [28, 27]. After the implementation of the RCOG recommendations in CUMH more women were diagnosed with GDM, even though a similar number of pregnancies were reported in the 2019 and 2020 study periods. There was no significant difference in maternal and obstetric characteristics of women diagnosed with GDM other than an increase in GDM positive women with a self-reported history of PCOS in the 2020 cohort. This correlates with current studies documenting increased rates of PCOS in the last decade due to improved diagnostic tests available [29]. However as with all self reported data, this can be subject to recall error and affect the quality of data collected [30].

To date, the OGTT has been the gold standard test for detecting prediabetes, GDM and Type 2 Diabetes Mellitus. All guidelines released by professional societies in Canada, Australia and the United Kingdom recommend a HbA_1c_ ≥ 41 mmol/mol to diagnose GDM during the COVID-19 pandemic [24] It is evident that HbA_1c_ use in pregnancy significantly increased during the pandemic in CUMH, where the current study was based. The use of GDM biomarkers (FPG, RPG and HbA_1c_) over the OGTT corresponded with an increase in diagnostic rate in primary and SCare upon adopting the new diagnostic guidelines with no significant differences seen in maternal and pregnancy characteristics. While the OGTT is recognised as a cumbersome test, it is worth noting that the HbA_1c_ test, whose use increased in the current study, also has limitations. In pregnancy there is an increased turnover of red blood cells, and in women diagnosed with anemia glucose intolerance can be underestimated if the HbA_1c_ test is used [31]. Also many studies suggests that trimester-specific reference intervals for HbA_1c_ during pregnancy should be used to manage pregnancy complicated by diabetes [32]. In response to the COVID-19 pandemic, revised testing strategies for GDM were different in different countries with focus on specificity over sensitivity and it is clear that no single strategy is universally acceptable in unprecedented times. The pandemic underscored the essential role of effective communication in implementing changes to GDM testing. It emphasised the need for clear communication channels to ensure that both GP and SCare providers are aligned in their approach, thereby enhancing the quality of patient care. Greater adherence to the RCOG guidelines was observed in SCare than in GP which corresponded to a greater increase in GDM diagnostic rate. Our study shows some limitations which should be considered. Data relating to maternal co-morbidities was incomplete for 2020 on our database therefore an extensive review could not be performed of how the RCOG guidelines affected pregnancy outcomes, also it is a single centre retrospective study. Future prospective studies would require those women that screened negative between gestation weeks 24–28 based on the Irish adaption of the RCOG guidance to be retested using the OGTT to determine potential missed cases of GDM. Further research is needed to compare the increased incidence of GDM using the RCOG recommendations with neonatal outcomes in order to identify the best option for possible future health emergencies.

## Conclusion

The COVID-19 pandemic emphasised the real concerns for pregnant individuals in relation to travel and the time spent in potentially infectious environments to perform an OGTT. The replacement of the OGTT during this uncertain time with other tests such as FPG, RPG and HbA_1c_ was a pragmatic approach, as an increase in GDM incidence was identified. HbA_1c_ test requests increased significantly in primary and SCare especially during the pandemic indicating adherence to the RCOG recommendations. Pregnancies with poor/uncontrolled glycaemia or at risk of glucose intolerance were identified, as an increase in GDM positivity rates were observed. This was favored over a decrease in GDM positivity rates and the potential to miss a GDM diagnosis during the pandemic positively impacting patient care. Careful review of these guidelines and their implications need to be further assessed to ensure hospitals are more knowledgeable for other future unpredictable events.

## Data Availability

The data presented in this study are available on request from the corresponding author due to ethical reasons.
